# Impact of a 12‐week High‐Intensity Interval Training With Spirulina Supplementation on Insulin Resistance‐Mediated by Apo‐A, ‐B, and ‐J in Men With Obesity

**DOI:** 10.1002/ejsc.12285

**Published:** 2025-03-23

**Authors:** Seyed Morteza Tayebi, Parisa Bagherian, Minoo Bassami, Aref Basereh, Somayeh Ahmadabadi

**Affiliations:** ^1^ Department of Exercise Physiology Faculty of Physical Education and Sports Sciences Allameh Tabataba'i University Tehran Iran; ^2^ Ph.D. Exercise Physiology Faculty of Sports Science Kharazmi University Tehran Iran; ^3^ Department of Physical Education and Sports Sciences Farhangian University Tehran Iran

**Keywords:** apolipoproteins, exercise training, HIIT, nutritional supplement, obesity, spirulina

## Abstract

Obesity is a significant public health issue associated with an elevated risk of chronic diseases. Discovering appropriate exercise interventions combined with beneficial herbal supplements has always been investigated. Hence, our study aimed to assess the impact of 12 weeks of high‐intensity interval training (HIIT) and spirulina supplementation on the apolipoproteins, insulin resistance, and body composition of men with obesity.

Forty‐four men with obesity (height: 168.42 ± 2.63 cm, body mass: 93.24 ± 2.23 kg; BMI: 32.89 ± 1.23 kg/m^2^; age: 25–40 years) were divided into four groups: control group (CG, *n* = 11), spirulina group (SG, *n* = 11), high‐intensity interval training group (HIITG, *n* = 11), and SG + HIIT group (*n* = 11). The intervention involved the daily administration of either spirulina or a placebo and HIIT three times a week for the training groups. Anthropometric indices, HOMA‐IR, insulin, and apolipoproteins (Apo‐A, Apo‐B, and Apo‐J) were measured before and after the 12‐week intervention.

Post‐intervention analysis indicated differences between the CG and the three interventional groups for percent body fat (%BF), insulin, HOMA‐IR, and apolipoproteins levels (*p* < 0.05). Spirulina supplementation with HIIT increased Apo‐A while causing decreases in Apo‐B and Apo‐J levels and improved body composition (weight, %fat), BMI, and HOMA‐IR (*p* < 0.05).

It can be concluded that the combined intervention of high‐intensity interval training (HIIT) and spirulina supplementation has a significant impact on mitigating obesity, managing weight, and reducing the risk of cardiovascular diseases.

**Summary**

- A 12‐week intervention combining high‐intensity interval training with spirulina supplementation demonstrated beneficial effects on an increase in APO‐A and a decrease in the levels of APO‐B and APO‐J.
- The manuscript highlights the potential benefits of spirulina supplementation, specifically its impact on apolipoproteins and body composition.
- The combined intervention of HIIT and spirulina supplementation has a significant impact on mitigating obesity, managing weight, and reducing the risk of cardiovascular diseases.

## 1 Introduction

Over the last 30 years, obesity has become a widespread health problem, caused by consuming more calories than are burned through physical activity, leading to excess fat buildup. This condition significantly raises the risk of chronic diseases such as cardiovascular disease, atherosclerosis, metabolic syndrome, and type 2 diabetes (Ogden et al. 2014; Tayebi, Eslami, et al. 2023; Tayebi, Saeidi, et al. 2023). Conversely, making lifestyle changes such as engaging in regular physical activity and incorporating herbal supplements into one's diet can significantly prevent and treat obesity and associated metabolic diseases.

Spirulina, a filamentous, unbranched alga belonging to the Oscillatoraceae algae family, is characterized by its blue‐green color. It has been suggested that spirulina may contribute to an increase in lean body mass due to its high protein content, particularly its abundance of branched‐chain amino acids such as leucine, valine, and isoleucine (Vieira Salla et al. 2016). Consequently, athletes have utilized spirulina to enhance their body composition and physical performance (Delfan et al. 2024; Hernández‐Lepe et al. 2019; Milasius et al. 2009). Research studies have demonstrated that spirulina significantly reduces plasma concentrations of total cholesterol, LDL‐C, and triglycerides while increasing HDL‐C levels (Serban et al. 2016; Supriya et al. 2023). The protein phycocyanin in spirulina reduces blood lipid levels by decreasing free radicals, preventing lipid peroxidation, and inhibiting NADPH expression. These effects are linked to the reduction of NADPH and NADH, which play key roles in metabolism (EL‐Sabagh et al. 2014).

HIIT training has been shown to increase insulin signaling and activity by reducing intracellular triglyceride accumulation and promoting fatty acid oxidation (Adams 2013). Lipolysis hormones released during HIIT training stimulate energy production and fat oxidation postexercise. Visceral fat tissue, which possesses a higher density of β‐adrenergic receptors, is particularly responsive to these hormones, resulting in a reduction in visceral fat deposition (Zouhal et al. 2008). Gillen et al. (2013) demonstrated that short‐term low‐volume HIIT is an effective strategy for improving body composition and muscle oxidative capacity in overweight or women with obesity (Gillen et al. 2013). Additionally, Andreato et al. (2019) reported that HIIT can serve as a secondary approach for managing obesity in adults (Andreato et al. 2019).

Apolipoproteins are proteins found on the surface of lipoproteins and are essential for regulating lipid transfer and metabolism. They are strongly linked to obesity and lipid‐related disorders. Apolipoprotein‐A (Apo‐A) is the main protein in HDL, making up about 70% of its structure (Greene et al. 2012). Numerous studies have demonstrated an inverse relationship between HDL levels and the risk of cardiovascular disease (Kunutsor and Laukkanen 2023; Razavi et al. 2023). Apolipoprotein B (Apo‐B) is part of the structure of LDL, and its mutation causes LDL to remain in the blood more than usual, and as a result, the possibility of atherosclerosis increases (Glavinovic et al. 2022). Additionally, conditions characterized by insulin resistance, such as obesity, metabolic syndrome, and type 2 diabetes, are associated with increased circulating levels of apolipoprotein J (Apo‐J). Apo‐J is significantly correlated with total cholesterol and LDL‐C levels (Rull et al. 2015; Tayebi, Eslami, et al. 2023; Tayebi, Saeidi, et al. 2023).

Physical activity has been shown to increase levels of Apo‐A and HDL while decreasing total cholesterol, LDL, and Apo‐B (Sarlak 2016a; Stanton et al. 2022). Weight loss in obese individuals has been associated with a reduction in Apo‐J levels (Arnold et al. 2011). Furthermore, supplementation with spirulina has demonstrated significant effects on lowering plasma concentrations of total cholesterol, LDL‐C, and triglycerides while increasing levels of HDL‐C (Serban et al. 2016). Despite the established benefits of HIIT and spirulina on metabolic health, their combined effects on apolipoproteins (Apo‐A, Apo‐B, and Apo‐J) in obese men remain unexplored. This gap is critical because apolipoproteins are key regulators of lipid metabolism and are strongly linked to obesity‐related disorders such as cardiovascular disease and type 2 diabetes. Investigating these effects could reveal new mechanisms and inform targeted strategies for managing obesity and its complications while contributing to the evidence base for nonpharmacological interventions like exercise and dietary supplements.

Given the lack of existing research on the effects of HIIT and spirulina supplementation on apolipoproteins (Apo‐A, Apo‐B, and Apo‐J) in men with obesity, this study aimed to investigate the potential impact of a 12‐week HIIT and spirulina supplementation regimen on these parameters, as well as insulin resistance and body composition in this specific population.

## 2 Materials and Methods

### 2.1 Participants

Forty‐four men with obesity (age = 25–40 years, height = 168.42 ± 2.63 cm, body mass = 93.24 ± 2.23 kg, BMI = 32.89 ± 1.23 kg/m2) were enrolled in the study and randomly assigned to one of the four groups: control group (CG, *n* = 11), spirulina group (SG, *n* = 11), high‐intensity interval training group (HIITG, *n* = 11), and SG + HIIT group (*n* = 11). Randomization was performed using a computer‐generated sequence by an independent researcher to ensure an equal distribution of baseline characteristics and minimize bias. Both participants and investigators were blinded to group assignments until the completion of the study.

Inclusion and exclusion criteria were applied to ensure the homogeneity of the sample, including (1) participants who were not physically active and not on a weight loss diet; (2) participants with a BMI > 30 kg/m^2^ and waist‐to‐height ratio (WHR) > 0.5 (3) they should not have any specific illness (e.g., cardiovascular disease and type 2 diabetes) or diet (e.g., maintaining usual diet and avoiding new supplements); and (4) those who missed more than three practice sessions were excluded from the study.

All participants provided informed consent in accordance with the ethical guidelines outlined in the Helsinki Declaration for Human Research. All protocols of this research were approved by the Institutional Review Board of Allameh Tabataba'i University (#ATU/MA/10/51100,1403).

### 2.2 Study Design

The current study is part of a multidimensional research project entitled Effect of a 12‐week HIIT with spirulina supplementation on cardiometabolic health aspects, approved by the research committee of the Allameh Tabataba'i University, Tehran, IRAN. [Grant Numbers: #ATU/MA/10/51100,1403]. The associated studies are Supriya et al. (2023) and Delfan et al. (2024).

## 3 Experimental Approach

Before commencing the training programs, participants underwent a familiarization session 1 week in advance to ensure an understanding of the study procedures, and measurements of height, mass, and body composition were recorded for each participant.

During the third session, all groups were provided with instructions on how to carry out the training protocols. This session also coincided with the measurement of body composition variables and peak oxygen uptake (VO_2_ peak). The 12‐week exercise training program, consisting of three sessions per week, was initiated in the two training groups (HIIT and SG + HIIT) following the baseline assessments. In contrast, participants in the control group (CG) were instructed to maintain their usual lifestyles throughout the study. To ensure consistency and minimize potential confounding factors, data collection took place at the same time of day, typically within a one‐hour window, and under uniform environmental conditions of approximately 20°C and a relative humidity of approximately 55%. Baseline measurements were obtained 48 h before the start of the training protocols, whereas posttests were conducted 48 h after the final session for all groups. Participants in the two training groups were instructed to maintain a consistent dietary regimen during the 48 h before the baseline assessment and the final measurements after training.

### 3.1 Nutrient Intake and Dietary Analysis

Nutrient intake and dietary analysis were assessed using 3‐day dietary records, including two weekdays and one weekend day, before and after the study. These records monitored alterations in customary dietary patterns over the study duration (Thomas et al. 2016). Each specific food item was entered in the Diet Analysis Plus version 10 software (Cengage, Boston, MA, USA), which calculated the total caloric intake and the proportion of energy derived from proteins, fats, and carbohydrates (Table 1).

**TABLE 1:** Mean (± SD) values of nutritional intake in the four study groups.

	CG		SG		HIIT		SG+HIIT	
	Pre	Post	Pre	Post	Pre	Post	Pre	Post
Energy (kcal/d)	2321 ± 47	2342 ± 56	2354 ± 101	2314 ± 100	2349 ± 117	2297 ± 117	2301 ± 126	2375 ± 157
CHO (g/d)	292 ± 30.4	295 ± 31.3	288.4 ± 25.1	278 ± 26.5	298 ± 41.6	270 ± 37.2	297 ± 39.6	269 ± 30.1
Fat (g/d)	91.2 ± 16.0	92 ± 19.8	95.5 ± 17.7	84 ± 16.2	94.4 ± 19.4	84.1 ± 15.2	918 ± 15.87	75.2 ± 18.3
Protein (g/d)	115 ± 17.0	119 ± 19.3	112 ± 15.5	105 ± 16.6	113 ± 13.8	103 ± 11.7	112 ± 11.5	101 ± 12.5

### 3.2 Measurement of the VO_2_ Peak and Body Composition

Body weight and stature were gauged using a calibrated scale and stadiometer (Seca, Germany). These measurements were then used to calculate body mass index (BMI) (kg/m^2^). Fat‐free mass (FFM) and fat mass (FM) were determined using a bio‐impedance analyzer (Medigate Company Inc., Dan‐dong Gunpo, Korea).

The evaluation of VO_2_ peak was conducted using a modified Bruce protocol in a controlled environment set at 21°C–23°C, consistent with established practices for overweight and obese cohorts (Hunter et al. 2008). This test consists of seven stages, each lasting 3 minutes. Throughout the test, a Metalyzer 3B‐Cortex was used to determine the VO_2_ peak, whereas an electrocardiogram recorded the heart rate. All tests were supervised by a sports medicine specialist or sports physiologist. The criteria for reaching peak oxygen consumption and terminating the activity included abnormal signs from the electrocardiogram, a rating of perceived exertion higher than 19, moderate to severe chest pain, a drop in blood pressure exceeding 10 mm Hg, dizziness, and paleness.

All test procedures for measuring VO_2_ peak were performed according to the guidelines of the American Association of Sports Medicine (ACSM) (Medicine 2013).

### 3.3 Exercise Protocol

The exercise intensity was gained based on individual VO_2_ peak values obtained from a 32‐min running exercise on a treadmill. Before each training session, participants engaged in a 5‐min warm‐up phase consisting of stretching, walking, and running. The exercise intensity for treadmill running was initially set at 65% of VO_2_ peak during the first week and progressively increased to 75% of VO_2_ peak in the following week. Throughout the weekly sessions, exercise intensities were adjusted to maintain the specified percentages of VO_2_ peak. High‐intensity interval training (HIIT) sessions commenced in the third and fourth weeks, with intervals of 4 min of running at 75% of VO_2_ peak followed by 4 min of passive recovery, totaling a 32‐min HIIT session. Subsequently, intervals were extended to 85% of VO_2_ peak for weeks 5, 6, and 7, with 4‐min active recovery intervals at 15% of their VO_2_ peak, maintaining the 32‐min HIIT duration. During weeks 8, 9, and 10, the intervals further increased to 90% of their VO_2_ peak with active recovery intervals at 30% of their VO_2_ peak for 4 min. The final weeks (11 and 12) involved intervals at 95% of VO_2_ peak with active rest at 50% of VO_2_ peak for 32 min. A 5‐min cool‐down at 50% of VO_2_ peak concluded each training session (Soltani et al. 2020). The control group maintained their usual activities without engaging in structured physical exercise.

## 4 Supplementation

Participants received spirulina capsules from Hellenic Spirulina Net (Production unit: Thermopigi, Sidorokastro, Serres, Greece), with a daily dosage of 6 g divided into morning and evening doses over 12 weeks (Mazokopakis et al. 2014). Both the CG and the TG were also provided with an equivalent quantity of placebo. Placebo capsules containing corn starch are colored and flavored with edible green dye to mimic the appearance of spirulina powder and further flavored with the essence of kiwi fruit. Adherence to the supplement regimen was considered satisfactory if consumption met or exceeded 80% of the prescribed amount.

### 4.1 Blood Markers

Fasting blood samples were collected from the right brachial vein of participants 48 h before and after the 12‐week training period. Participants fasted for 12 h before blood sampling to reduce dietary effects on lipid and glucose levels (Supriya et al. 2023). Morning samples were taken to control for diurnal variations in hormones and lipids. This timing, supported by prior studies, ensures reliable metabolic marker measurements in obese individuals (Sarlak 2016a; Supriya et al. 2023). Plasma samples were prepared using tubes containing EDETA and centrifuged for 10 min at 3000 before storage at −80°C. Plasma Apo‐A and Apo‐B levels were measured by the immunoturbidimetric method using the quantitative detection kit of Bionic Company, whereas Apo‐J levels were measured using an ELISA kit (Boster Biological Technology, Pleasanton, CA, USA). Insulin levels were determined using an ELISA kit (Demeditec, Germany), with a sensitivity of 1 ng/mL and within coefficient variations of 5.1%–8.4%. Insulin resistance was calculated with the homeostasis model assessment of insulin resistance (HOMA‐IR), using the equation (fasting insulin in μU/mL × fasting glucose in mmol/L)/22.5. All procedures were conducted under standardized conditions to ensure consistency in data collection.

## 5 Statistical Methods

The statistical methods employed in this study involved both descriptive and inferential analyses, with the distribution of data assessed using the Shapiro–Wilk test. The univariate ANCOVA test was utilized to ascertain differences between groups while adjusting for pretraining variables, with statistical significance set at *p* < 0.05. All statistical analyses were conducted using SPSS (Version 25.0; IBM SPSS Inc., Chicago, IL, USA).

## 6 Results

### 6.1 Apolipoproteins

Table 2 shows the mean and standard deviation of the research variables. The results of the univariate ANCOVA test showed a significant difference between groups in Apo‐A, Apo‐B, and Apo‐J while adjusting for the pretraining (Table 2). Bonferroni's correction *post hoc* test for Apo‐A showed a significant difference in SG (Mean Difference (MD): 26.89, 95% CI for MD: 8.90, 44.88, *p* = 0.002), HIIT (MD: 38.05, 95% CI for MD: 19.95, 56.14, *p* < 0.001), and HIIT + SG (MD: 43.14, 95% CI for MD: 61.04, 25.27, *p* < 0.001) compared to CG. No difference was found between the other groups (*p* > 0.05).

**TABLE 2:** The mean and standard deviation of the research variables.

Variables		Groups				Univariate ANCOVA		
		CG	SG	HIIT	SG + HIIT	F	*p*	η^2^
Apo‐A (mg/dL)	Pretraining	184.00 ± 13.80	188.49 ± 11.49	189.99 ± 14.31	185.19 ± 14.87	0.103	0.750	0.001
	Posttraining	186.89 ± 16.10	214.05 ± 19.46	225.29 ± 12.75	230.10 ± 12.33	16.57	0.001*	0.560
Apo‐B (mg/dL)	Pretraining	137.48 ± 14.81	128.07 ± 25.33	130.75 ± 16.97	139.33 ± 12.66	0.024	0.877	0.001
	Posttraining	149.28 ± 19.98	113.25 ± 9.85	103.42 ± 15.62	91.03 ± 17.06	26.11	0.001*	0.668
Apo‐J (mg/dL)	Pretraining	133.43 ± 11.06	140.13 ± 10.35	132.34 ± 18.23	139.20 ± 16.47	3.04	0.089	0.072
	Posttraining	142.17 ± 8.43	119.17 ± 10.07	98.87 ± 8.95	95.15 ± 9.54	16.57	0.001*	0.827
Body weight (kg)	Pretraining	93.88 ± 2.05	92.92 ± 2.86	92.42 ± 1.94	93.77 ± 1.92	1.87	0.179	0.019
	Posttraining	93.01 ± 2.20	90.86 ± 1.82	88.83 ± 1.66	87.08 ± 2.15	18.62	0.001*	0.578
BMI (kg/m2)	Pretraining	32.90 ± 1.44	32.48 ± 1.40	33.04 ± 1.29	33.14 ± 0.76	38.74	0.001*	0.354
	Posttraining	32.60 ± 1.50	31.75 ± 0.83	31.76 ± 1.28	30.78 ± 0.99	10.55	0.001*	0.289
Fat percentage (%)	Pretraining	30.55 ± 1.01	29.66 ± 0.80	29.91 ± 1.14	30.40 ± 1.06	0.138	0.713	0.009
	Posttraining	30.56 ± 1.13	28.54 ± 0.81	27.26 ± 0.81	26.81 ± 1.01	33.77	0.001*	0.0721
Fasting blood sugar (mg/dL)	Pretraining	96.45 ± 13.10	98.99 ± 10.72	99.28 ± 5.73	101.63 ± 7.14	4.03	0.052	0.033
	Posttraining	93.19 ± 8.91	84.77 ± 4.50	74.09 ± 5.44	71.51 ± 7.72	26.42	0.001*	0.648
Insulin	Pretraining	18.81 ± 0.67	18.81 ± 0.76	18.84 ± 0.40	19.13 ± 0.50	0.30	0.583	0.009
	Posttraining	19.13 ± 0.58	17.60 ± 0.52	16.13 ± 0.43	15.51 ± 0.56	99.39	0.001*	0.884
Insulin resistance	Pretraining	4.49 ± 0.70	4.59 ± 0.46	4.61 ± 0.25	4.80 ± 0.39	2.14	0.151	0.009
	Posttraining	4.28 ± 0.31	3.68 ± 0.26	2.95 ± 0.27	2.74 ± 0.32	67.19	0.001*	0.830

*Statistically significant.

Bonferroni's correction *post hoc* test for Apo‐B showed a significant difference in SG compared to HIIT+SG (MD: −22.47, 95% CI for MD: −41.55, −3.38, *p* = 0.018). SG (MD: −35.82, 95% CI for MD: −54.76, −16.88, *p* < 0.001), HIIT (MD: −9.89, 95% CI for MD: −28.51, 8.73, *p* < 0.001), and HIIT + SG (MD: −58.29, 95% CI for MD: −76.90, −39.68, *p* < 0.001) showed a significant difference compared to CG. No difference was found between the other groups (*p* > 0.05).

Bonferroni's correction *post hoc* test for Apo‐J showed a significant difference in SG compared to HIIT (MD: −21.64, 95% CI for MD: −32.19, −11.09, *p* < 0.001) and HIIT + SG (MD: −24.18, 95% CI for MD: −34.52, −13.83, *p* < 0.001). Also, SG (MD: −21.83, 95% CI for MD: −32.33, −11.34, *p* < 0.001), HIIT (MD: −43.48, 95% CI for MD: −53.83, −33.13, *p* < 0.001), and HIIT+SG (MD: −46.02, 95% CI for MD: −56.47, −35.36, *p* < 0.001) showed a significant difference compared to CG. There was no difference between the HIIT and HIIT + SG groups (MD: −2.53, 95% CI for MD: −13.03, 7.97, *p* = 1.00).

## 7 Body Composition, Fasting Blood Glucose, Insulin, and Insulin Resistance

Also, the univariate ANCOVA test results showed a significant difference between groups after 12 weeks of training in body weight, BMI, fat percentage, fasting blood glucose, insulin, and insulin resistance while adjusting for the pretraining (Table 2).

Bonferroni's correction *post hoc* test for body weight showed a significant difference in HIIT + SG compared to CG (MD: −5.91, 95% CI for MD: −8.14, −3.67, *p* < 0.001) and SG (MD: −3.94, 95% CI for MD: −6.19, −1.68, *p* < 0.001). HIIT (MD: −3.90, 95% CI for MD: −6.20, −1.60, *p* < 0.001) showed a significant difference compared to CG. No difference was found between the other groups (*p* > 0.05).

Bonferroni's correction *post hoc* test for fat percentage showed a significant difference in SG compared to CG (MD: −2.07, 95% CI for MD: −3.23, −0.92, *p* < 0.001), HIIT (MD: −1.26, 95% CI for MD: −2.36, −0.16, *p* = 0.023), and HIIT+SG (MD: −1.68, 95% CI for MD: −2.82, −0.55, *p* = 0.002). Also, HIIT (MD: −3.33, 95% CI for MD: −4.46, −2.21, *p* < 0.001) and HIIT+SG (MD: −3.76, 95% CI for MD: −4.86, −2.66, *p* < 0.001) showed a significant difference compared to CG. There was no difference between the HIIT and HIIT+SG groups (MD: −0.42, 95% CI for MD: −1.54, 0.68, *p* = 1.00).

Bonferroni's correction *post hoc* test for BMI showed a significant difference in HIIT+SG compared to CG (MD: −1.98, 95% CI for MD: −2.95, −1.01, *p* < 0.001), SG (MD: −1.41, 95% CI for MD: −2.39, −0.42, *p* = 0.003), and HIIT (MD: −1.05, 95% CI for MD: −2.01, −0.08, *p* = 0.035). No difference was found between the other groups (*p* > 0.05).

Bonferroni's correction *post hoc* test for insulin showed a significant difference in SG compared to CG (MD: −1.52, 95% CI for MD: −2.12, −0.91, *p* < 0.001), HIIT (MD: −1.47, 95% CI for MD: −2.08, −0.87, *p* < 0.001), and HIIT+SG (MD: −2.06, 95% CI for MD: −2.68, −1.45, *p* < 0.001). HIIT (MD: −2.99, 95% CI for MD: −3.60, −2.35, *p* < 0.001) and HIIT+SG (MD: −3.59, 95% CI for MD: −4.20, −2.97, *p* < 0.001) showed a significant difference compared to CG. There was no difference between the HIIT and HIIT+SG groups (MD: −0.59, 95% CI for MD: −1.20, 0.02, *p* = 0.083).

Bonferroni's correction *post hoc* test for insulin resistance showed a significant difference in SG compared to CG (MD: −0.61, 95% CI for MD: −0.93, −0.21, *p* < 0.001), HIIT (MD: −0.73, 95% CI for MD: −1.06, −0.41, *p* < 0.001), and HIIT+SG (MD: −0.97, 95% CI for MD: −1.30, −0.64, *p* < 0.001). HIIT (MD: −1.34, 95% CI for MD: −1.67, −1.01, *p* < 0.001) and HIIT+SG (MD: −1.58, 95% CI for MD: −1.92, −1.24, *p* < 0.001) showed a significant difference compared to CG. There was no difference between the HIIT and HIIT+SG groups (MD: −0.23, 95% CI for MD: −0.56, 0.09, *p* = 0.083).

## 8 Relationship Between Apolipoproteins and Body Composition, Fasting Blood Glucose, Insulin, and Insulin Resistance

**FIGURE 1:**
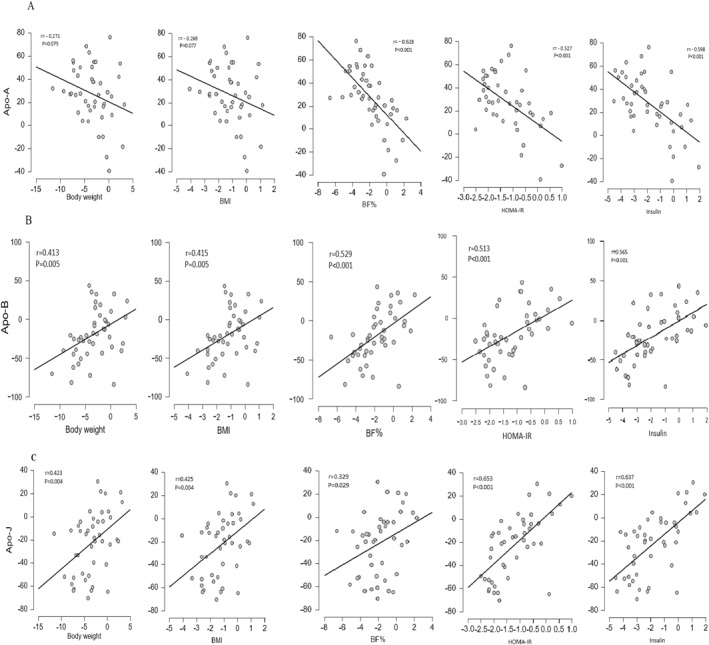
Correlation between changes in apolipoproteins compared with change in body composition (body fat %, body weight, and BMI), fasting blood glucose, insulin, and HOMA‐IR. Correlation between change in (A) Apo‐A, (B) Apo‐B, and (C) Apo‐J and change in body composition (body fat %, body weight, and BMI), fasting blood glucose, insulin, and HOMA‐IR. The *r* value is based on Spearman correlation. *p* < 0.05 is considered significant.

The relationship between changes in the apolipoprotein level and body composition, fasting blood glucose, insulin, and insulin resistance was also assessed (Figure 1). The results of the Spearman's correlation analysis revealed a statistically significant, strong negative correlation between Apo‐A and body fat percentage (*r* = ‐0.628, *p* < 0.001), insulin resistance (*r* = ‐0.527, *p* < 0.001), and fasting insulin concentrations (*r* = −0.598, *p* < 0.001). Conversely, a statistically significant positive correlation was observed between Apo‐B and body weight (*r* = 0.413, *p* < 0.001), body mass index (BMI) (*r* = 0.415, *p* < 0.001), body fat percentage (*r* = 0.529, *p* < 0.001), insulin resistance (*r* = 0.513, *p* < 0.001), and fasting insulin concentrations (*r* = 0.565, *p* < 0.001). Similarly, a positive and significant correlation was found between Apo‐B and body weight (*r* = 0.423, *p* < 0.001), BMI (*r* = 0.425, *p* < 0.001), body fat percentage (*r* = 0.329, *p* < 0.001), insulin resistance (*r* = 0.653, *p* < 0.001), and fasting insulin concentrations (*r* = 0.637, *p* < 0.001).

Apo‐A indicates apolipoprotein A‐I; Apo‐B, apolipoprotein B, Apo‐J, apolipoprotein J; and HOMA‐IR, homeostatic model assessment of insulin resistance.

## 9 Discussion

The study observed that interventions such as 12 weeks of high‐intensity interval training (HIIT), spirulina supplementation, and a combination of HIIT with spirulina supplementation resulted in an increase in apolipoprotein‐AI (Apo‐AI) levels and a decrease in apolipoprotein‐B (Apo‐B) and apolipoprotein‐J (Apo‐J) levels in men with obesity compared to the control group. Higher Apo‐A, a key component of HDL, suggests improved cardiovascular health and reduced atherosclerosis risk (Boekholdt et al. 2013). Lower Apo‐B, linked to LDL, indicates decreased cardiovascular risk, whereas reduced Apo‐J levels may reflect better insulin sensitivity and lipid metabolism (Rull et al. 2015; Tayebi, Eslami, et al. 2023; Tayebi, Saeidi, et al. 2023). These findings align with previous studies on spirulina (Bohórquez‐Medina et al. 2021; Supriya et al. 2023) and exercise (Sarlak 2016a; Stanton et al. 2022) benefits and highlight the potential of combining HIIT and spirulina for managing obesity‐related metabolic disorders. These findings align with previous studies (Holme et al. 2007; Parsa et al. 2021; Sarlak 2016b; Soleimani et al. 2023), although some research has indicated that apolipoprotein levels do not change with exercise training (Feyzi et al. 2023; Ghorbanian et al. 2015; Shearman et al. 2010). For instance, Feyzi et al. (2023) demonstrated that 8 weeks of HIIT did not induce changes in apolipoprotein levels (Feyzi et al. 2023), whereas other studies showed that after 8 weeks of high‐intensity interval endurance training, Apo‐A levels remained unchanged (Ghorbanian et al. 2015; Shearman et al. 2010). These findings suggest that the intensity and volume of exercise play a significant role in influencing blood lipid profiles and apolipoprotein levels. Shearman et al. (2010) conducted a study using an endurance training protocol with an intensity of 50%–60% of the maximum heart rate in adult men and showed that after 6 weeks, the changes in Apo‐A were negligible (Shearman et al. 2010). However, with the continuation of training after 14 weeks, the changes were significant. Moreover, it has been shown that high‐intensity and high‐volume training have a more pronounced impact on the concentration of these factors compared to low or moderate intensity and low‐volume training (Williams et al. 1992).

This study also revealed that after 12 weeks of HIIT, there was a significant change in apolipoprotein concentration levels (Apo‐A↑, Apo‐B↓, and Apo‐J↓). This effect is attributed to the increase in mitochondrial content resulting from HIIT, leading to improved skeletal muscle mitochondria function and increased fat oxidation (Little et al. 2011). Additionally, HIIT induces the release of lipolytic hormones such as growth hormone and epinephrine, leading to greater energy production and increased fat oxidation postexercise. Since visceral fat tissue is more sensitive to these hormones due to having more β‐adrenergic receptors, it therefore results in a desirable reduction of visceral fat.

Conversely, spirulina supplementation was found to increase Apo‐A levels and reduce Apo‐B and Apo‐J levels compared to the control group. Spirulina supplementation also significantly reduced triglyceride and total cholesterol levels in individuals with metabolic syndrome or obesity (Bohórquez‐Medina et al. 2021; Supriya et al. 2023). The hypolipidemic effects of spirulina are attributed to its main constituent, phycocyanin, which regulates plasma lipid concentrations by inhibiting free radical production, lipid peroxidation, suppressing the expression of nicotinamide adenine dinucleotide phosphate (NADPH), and enhancing the activity of glutathione peroxidase and superoxide dismutase (EL‐Sabagh et al. 2014). Additionally, spirulina's high phenylalanine content significantly contributes to appetite reduction (Mridha et al. 2010).

The current study revealed a significant association between apolipoproteins and various metabolic parameters such as body weight, BMI, body fat percentage, blood glucose, insulin levels, and insulin resistance. Notably, in therapeutic interventions accompanied by weight loss, which lead to improved insulin sensitivity, the circulating levels of Apo‐J also decrease (Seo et al. 2018), and these results are consistent with the findings of the present study, which showed a strong positive correlation between Apo‐B and Apo‐J with weight loss. Previous studies have also highlighted the link between changes in anthropometric measures like body weight, BMI, and body fat percentage with alterations in apolipoprotein levels (Ghorbanian et al. 2015; Sarlak 2016a). Body weight, BMI, and (BF %) are factors used as indicators of obesity. Elevated body weight, BMI, and body fat are commonly associated with an unfavorable lipid profile, characterized by reduced HDL cholesterol and increased levels of triglycerides, LDL cholesterol, Apo‐B, and Apo‐J (Nicklas et al. 1997). A sedentary lifestyle and lack of physical activity contribute to dyslipidemia, obesity, and overweight, increasing the risk of cardiovascular diseases through factors such as high waist‐to‐hip ratio, lipid disorders, inappropriate lipid concentrations, and imbalanced apolipoprotein levels A, apolipoprotein B, and Apo‐B/Apo‐A ratio, which are major factors associated with cardiovascular diseases (Delfan et al. 2024; Ghorbanian et al. 2015; Hosseinian et al. 2016).

## 10 Limitations

Despite the valuable insights gained from this study, certain limitations should be acknowledged. First, the study lacked control over the genetic variations among participants, which could influence individual responses to the interventions. Second, external factors such as extracurricular activities outside the prescribed training regimen were not closely monitored, potentially affecting the outcomes. Lastly, the study was unable to regulate participants' motivation levels and adherence to the training program, which may have influenced the results.

## 11 Conclusion

In conclusion, the 12‐week intervention combining high‐intensity interval training with spirulina supplementation demonstrated beneficial effects on apolipoprotein levels, an increase in APO‐A and a decrease in the levels of APO‐B and APO‐J, subsequently leading to weight loss, reduced body fat percentage, insulin levels, insulin resistance, and BMI in overweight men. The findings underscore the potential of this combined approach in mitigating obesity, managing weight, and reducing cardiovascular disease risk. However, individual responses may vary based on factors such as age, sex, baseline fitness level, and genetic predisposition. For example, individuals with severe obesity or preexisting cardiovascular conditions may experience different outcomes, and further research is needed to explore these effects in diverse populations.

## Author Contributions

SMT was responsible for this study and also designed it. PB wrote the first draft. MB cooperated with training sessions. MB and PB helped with data interpretation. AB helped with testing and training protocol. SA analyzed data and helped in revising the draft.

## Ethics Statement

All procedures performed in studies involving human participants were under the ethical standards of the institutional and national research committee and with the 1964 Helsinki Declaration and its later amendments or comparable ethical standards. All protocols of this research were approved by the Institutional Review Board of Allameh Tabataba'i University (#ATU/MA/10/51100,1403).

## Conflicts of Interest

The authors declare no competing interests.

## Data Availability

All data generated or analyzed during this study are included in this published article.
